# CASE REPORT Type II Metacarpal Hands: Reconstruction Planning Revisited

**Published:** 2010-07-16

**Authors:** Theresa Y. Wang, Ming-Chung Yeh, Yu-Te Lin, Fu-Chan Wei

**Affiliations:** ^a^Division of Plastic Surgery, University of Pennsylvania, Philadelphia, Pennsylvania; ^b^Department of Plastic and Reconstructive Surgery, Chang Gung Memorial Hospital, Taipei, Taiwan

## Abstract

**Objective:** Successful reconstruction of the metacarpal hand requires thorough evaluation and careful surgical planning. Effective transplantation involves 3 main considerations: residual hand function, functional needs and desires of the patient, and optimal surgical management to maximize outcome and minimize patient morbidity. **Methods:** The following is a clinical example of the metacarpal hand in which the patient underwent initial reconstruction at an outside hospital and was referred to our institution. This demonstrates how the initial planning and surgical management could have been further optimized to minimize functional deficits and donor-site morbidities as well as reduce the number of subsequent revisional surgeries and rehabilitation time. **Results:** Several important points in metacarpal hand reconstruction are described given specific level of amputation and residual function after the injury—the timing and sequence of operative strategy depending on the type of injury, the selection of donor-site digit transfers, and the overall treatment strategies for thumb and finger reconstruction. **Conclusion:** It is important to follow proper treatment algorithms in order to determine appropriate timing and sequence of toe-to-digit transfers, multi-stage versus 1-stage, as well as define the reconstructive goal to achieve a tripod pinch for a unilateral or a dominant hand injury or a pulp-to-pulp opposition for nondominant injury in bilateral cases. If adequate planning is performed, unnecessary and additional surgical procedures as well as increased patient suffering and prolonged rehabilitation time can be prevented or optimized.

*Metacarpal hand* refers to the amputation of fingers, with or without thumb, resulting in the loss of prehensile ability, which is essential for basic hand function.^1^,^2^ A definition of the metacarpal hand along with guidelines for the selection of the proper treatment algorithms with various toe transfers has been well described.^2^,^3^ It is classified into 2 categories: type I and type II. Type I metacarpal hand consists of amputations of all fingers proximal to the middle part of the proximal phalanx with either a normal thumb or one amputated distal to the interphalangeal joint and is further subtyped into 3 groups on the basis of the location of the finger amputations in relation to the metacarpophalangeal joint. Type II also comprises finger amputations proximal to the proximal phalanx, but along with which the thumb is also amputated proximal to the interphalangeal joint. It is subdivided into 4 groups focusing on the different levels of thumb amputation as well as the status of the thenar musculature and basal joint mobility (Table 1). This method of classification takes into consideration the specific type of injury and the associated treatment strategies with positive predicted outcomes.

Mutilating hand injuries pose a major reconstructive challenge to the surgeon. Much has been solved with breakthroughs in toe-to-hand transfers; microsurgery remains the foremost effective reconstructive method for these types of injuries.^2^^-^7 Despite well-established treatment protocols for toe-to-finger transfers, surgeons are still confronted with a formidable task. Transplantations are complex and time-consuming and in order to achieve optimal results, the previously proposed guidelines need to be taken into careful consideration when planning the operative approach. Effective metacarpal hand reconstruction requires 3 main considerations: the residual hand functions, functional needs and desires of the patient, and the most appropriate surgical management to maximize outcome and minimize morbidity to the patient. Careful evaluation and surgical planning will provide appropriate timing and sequence of toe-to-digit transfers, multi-stage versus 1-stage as well as define a suitable reconstructive goal to achieve a tripod pinch for a unilateral or a dominant hand injury versus pulp-to-pulp opposition for nondominant injury in bilateral cases.

The following is a clinical case of the metacarpal hand in which the patient underwent initial reconstruction at an outside hospital and was subsequently referred to our institution. This demonstrates how the initial planning and surgical management resulted in functional deficits and donor-site morbidities that could have been further optimized from the beginning. It gives us the opportunity to revisit and reflect on the several important aspects essential for an optimal reconstruction in such a mutilating hand injury. This case demonstrates that through thoughtful consideration in the initial preoperative assessment and preparation, surgical complexity and patient morbidity as a result of additional surgical procedures and prolonged rehabilitation time can be decreased.

## CASE REPORT

This 36-year-old right-hand-dominant man was referred to our institution (6/27/03) for further reconstruction after initial management at an outside hospital. He sustained a severe crush injury to his right hand several months prior (2/22/03) from factory machinery that resulted in a type IIA metacarpal hand with thumb amputation at the metacarpophalangeal joint and total amputation of all 4 fingers at the proximal phalanx. At the time of injury, a free latissimus dorsi myocutaneous flap was transferred for soft-tissue coverage of the dorsum of the hand (2/22/03). The right great toe was disarticulated at the metatarsophalangeal joint and transplanted (3/6/03) to reconstruct the right thumb.

**Figure 1:**
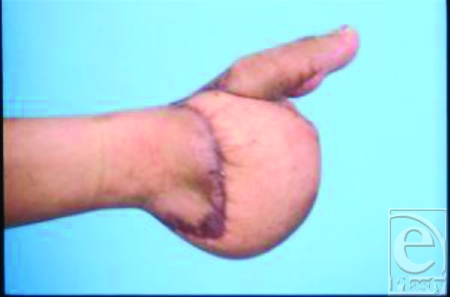
Initial presentation to our institution (6/27/03) post—latissimus dorsi myocutaneous flap and right great toe-to-thumb transplantation. The thumb was malpositioned with excessive length.

**Figure 2:**
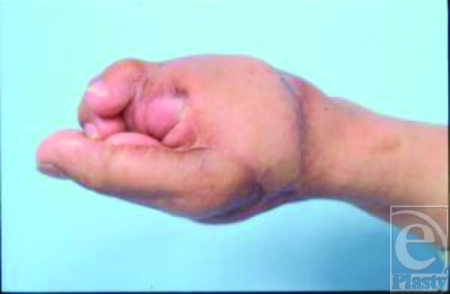
Appearance after second and third toe-to-finger transplantation (8/6/03). Given the initial length and positional discrepancies of the transplanted thumb, the patient is unable to use the reconstructed fingers to form an effective tripod pinch.

On presentation to our hospital, our initial evaluation produced several major findings. The latissimus dorsi free flap was bulky and had already sacrificed 1 set of potential vessels from the hand for any future microsurgical toe transfer. The new transplanted thumb looked too long, was malpositioned, and was rotated laterally (Fig 1). This patient had adequate thenar muscle function in his right hand. The reconstructed thumb had fused metacarpo- to metatarsophalangeal joint. Furthermore, as a result of the right great toe transfer through the metatarsophalangeal joint, the patient was unable to walk without shoes (Fig 2).

**Figure 3:**
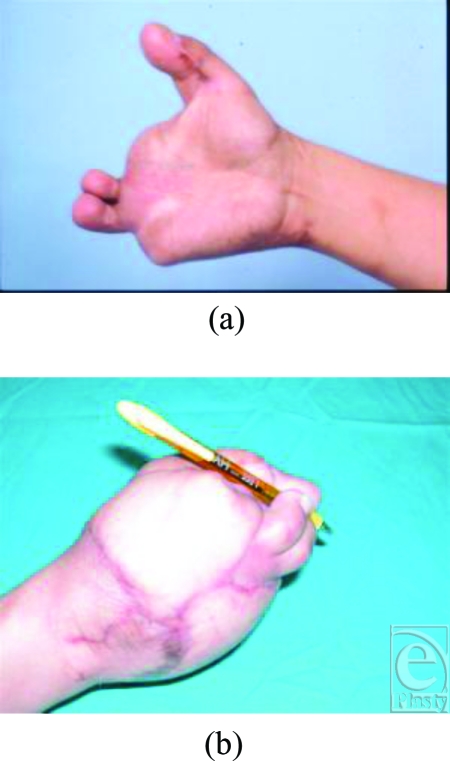
Appearance and function after corrective osteotomy of the transplanted thumb and after a series of debulking and revisioning surgeries (2/18/04 to 9/29/04). The patient now has the correct length to produce a tripod pinch.

After thorough discussions with the patient, our plan was to reconstruct a functional tripod pinch in this unilateral hand injury with a combined left second and third toe transfer to reconstruct the third and fourth fingers (8/6/03). Given that the right great toe was already used in the thumb transfer, we opted to use the left second and third toe. The postoperative course was smooth, but the length and positional discrepancy in the previously transplanted great toe-to-thumb prevented effective opposition with the newly transferred second and third toe fingers (Fig 3), rendering the thumb transplant less useful. We then completed a corrective shortening and rotational osteotomy of the transplanted thumb (2/18/04); the first metacarpal phalanx and the proximal phalanx of the transplanted great toe were both reduced and fused. A series of further surgical enhancements were completed (between 8/11/04 and 9/29/04) to provide improvements in function and cosmesis. These included revisions of unsightly scars, scar contracture releases, debulking of the latissimus dorsi flap, reduction of bony prominences, and excision of redundant skin. In addition, central pulp debulking of the right great toe-to-thumb transplantation was performed (9/29/04). After our sequential reconstruction and revision efforts, the patient can now use his right hand effectively in a tripod pinch (2/18/05) (Figs 3a and 3b).

## DISCUSSION

The general algorithmic scheme to treating metacarpal hands begins with the careful and thorough assessment of the amputated anatomy as well as the residual function of the hand immediately after the injury. It is during the initial evaluation of the injury that the planning of toe-to-finger transfers should begin. Several major components of the management protocol are integral to successful reconstruction.^2^,^4^ It is crucial to avoid excessive shortening of the skeleton, the tendon as well as neurovascular bundles to allow flexibility in the subsequent stages of reconstruction.^2^,^8^ If soft-tissue coverage of the wound is needed, the pedicled groin flap is of choice. Local flaps should be avoided to prevent creation of further scarring which increases the difficulty of future toe-transfer surgery and should also be avoided because it sacrifices a set of vessels, which can be utilized as recipient vessels in the future toe transplantation. The pedicled groin flap should be designed in an adequate or even redundant manner. The redundant skin can be a helpful contributor later on—covering the lateral aspects of the toe transfers, creating an acceptable web space in the hand, and minimizing the amount of skin necessary in the toe harvest thereby allowing for primary donor-site closure and decreased morbidity.^2^

When planning the reconstructive strategy, the proposed classification and surgical management protocol should be observed. For the unilateral type II metacarpal hand, regardless of hand dominance, the aims of the reconstruction are to provide a thumb and 2 fingers to achieve a tripod pinch instead of a thumb and 1 finger for pulp-to-pulp or pulp-to-side pinch. The reconstruction of 2 adjacent digits will produce such a tripod pinch that improves lateral stability and provides a wider and stronger hand for grasping objects.^5^,^9^^-^12 The residual function of the thenar muscles and the motion of the basal joint are of paramount importance in determining the correct sequence of surgical management.^10^ With adequate thenar musculature function (type IIA and IIB) and basal joint motion, appropriate planning and management would be 1-stage, simultaneous reconstruction of both fingers and thumb, with combined second and third toes from the right foot and the great toe from the left foot. The combined toe transfer is preferred for adjacent finger amputations that are proximal to the web space. If distal to the web space, 2 single-finger transfers are more suitable to prevent a syndactylous appearance.^13^ However, if the thenar muscle function or basal joint motion were inadequate or nonexistent (type IIC), then a multi-stage reconstruction should be planned with finger reconstruction preceding that of the thumb.^2^,^14^ In the meantime, a prosthetic thumb post can be used to help the surgeon determine more precisely the ideal length and position for the future thumb reconstruction with great toe transfer.^2^

Regardless of which thumb is to be reconstructed, the first choice should always be the left great toe in order to not hinder functional capabilities in everyday activities such as driving. When the great toe is harvested, approximately 1 cm of the proximal phalanx should be preserved because it is important for the push-off function of the foot.^2^ Furthermore, the great toe is usually 1 to 1.5 cm longer than the thumb from the levels of the metatarso- and the metacarpophalangeal joints. Therefore, the great toe is harvested from the proximal phalanx leaving behind 1 cm of its base and consequently can compensate for thumb amputations up to the metacarpal neck level. Great toe harvest should avoid metatarsophalangeal joint disarticulation and transmetatarsal amputations to minimize donor-site morbidity and preserve maximal foot functions. If needed, metacarpal stumps can also be augmented to the adequate length with nonvascularized bone graft interposed between the transferred great toe and the metacarpal stump.^10^ It should also be cautioned that the second and third toes are inherently shorter in length compared to fingers. Therefore the length of the thumb reconstruction should be planned at a shorter length in order to complement and allow an effective tripod pinch; the thumb cannot be restored to its original length if the goal is to achieve function.

Effective and functional reconstruction of the metacarpal hand begins with careful assessment and preparation. Errors leading to increased number of surgical procedures, increased patient suffering, and prolonged rehabilitation process can be prevented if adequate planning is achieved. In an already challenging reconstructive undertaking, thorough planning and surgical management can help to yield optimal results with minimal morbidity.

## Figures and Tables

**Table 1:** Unilateral type II metacarpal hand treatment algorithm

	Type IIA	Type IIB	Type IIC	Type IID
Level of amputation and residual functions	Distal to metacarpal neck	Proximal to metac arpal neck with intact thenar musculature	Any level with inadequate thenar musculature	Any level with damaged carpometacarpal joint
Strategy: sequence and timing	One-stage: simultaneous thumb and fingers transfers	One-stage: simultaneous thumb and- fingers transfers	Two-stage: fingers-preceding thumb transfers	Two-stage: fingers preceding thumb post
Thumb reconstruction	Trimmed left great-toe transfer	Lengthening with bone graft followed by trimmed left great toe transfer	Trimmed left great toe transfer and tendon transfer to restore opposition	Same as in IIA and IIB with aim to reconstruct an immobile thumb post
Finger reconstruction	Combined right 2, 3toes or 3, 4 toes	Combined right 2, 3 toes or 3, 4 toes	Combined right 2, 3 toes or 3, 4 toes	Combined right 2, 3 toes or 3, 4 toes
